# A Sheep Model for Cancellous Bone Healing

**DOI:** 10.3389/fsurg.2014.00037

**Published:** 2014-09-08

**Authors:** Angad Malhotra, Matthew Henry Pelletier, Yan Yu, Chris Christou, William Robert Walsh

**Affiliations:** ^1^Surgical and Orthopedic Research Laboratories, Prince of Wales Clinical School, University of New South Wales, Sydney, NSW, Australia

**Keywords:** ovine, sheep, model, bone healing, micro ct, age, hard tissue, histology

## Abstract

Appropriate well-characterized bone defect animal models remain essential for preclinical research. This pilot study demonstrates a relevant animal model for cancellous bone defect healing. Three different defect diameters (8, 11, 14 mm) of fixed depth (25 mm) were compared in both skeletally immature (18-month-old) and aged sheep (5-year-old). In each animal, four defects were surgically created and placed in the cancellous bone of the medial distal femoral and proximal tibial epiphyses bilaterally. Animals were euthanized at 4 weeks post-operatively to assess early healing and any biological response. Defect sites were graded radiographically, and new bone formation quantified using μCT and histomorphometry. Fibrous tissue was found within the central region in most of the defects with woven bone normally forming near the periphery of the defect. Bone volume fraction [bone volume (BV)/TV] significantly decreased with an increasing defect diameter. Actual BV, however, increased with defect diameter. Bone ingrowth was lower for all defect diameters in the aged group. This pilot study proposes that the surgical creation of 11 mm diameter defects in the proximal tibial and distal femoral epiphyses of aged sheep is a suitable large animal model to study early healing of cancellous bone defects. The refined model allows for the placement of four separate bone defects per animal and encourages a reduction in animal numbers required for preclinical research.

## Introduction

Bone grafts remain essential for the augmentation and repair of bone defects arising from trauma, resection, non-unions, and osteolysis. As the pursuit for alternatives to autograft continues, appropriate, reproducible, and well-characterized animal models remain essential tools for preclinical research. With any animal research, the appropriate use of research animals while maintaining their well-being should remain a priority (1). This study presents a refined animal bone healing model accordingly. Animal models need to satisfy both experimental and clinical goals and should demonstrate relevant and achievable treatment outcomes. Appropriate bone healing models should therefore aim to replicate clinical scenarios in which a realistic healing rate is reproduced in the animal model.

While the bone healing of many animal species is recognized as faster than in humans, sheep are considered to have a comparable rate to that of humans (2) and have been previously established as useful models for human bone turnover and remodeling activity (3). Therefore, this study investigated ovine bone defect healing at 4 weeks. In sheep, the growth of the distal femur and proximal tibia cease at 18–26 months of age, with cancellous bone defects of 10 mm in diameter previously reported as critical (2, 3); however, it has not been established whether this definition can be translated to aged animals. Healthy, normal, and robust animals are commonly used as bone healing models with their experimental outcomes commonly being translated to unhealthy, aged humans (1). Aged animals are likely to have a lower occurrence of spontaneous bone remodeling than that typically seen in younger animals. This effect has the potential to mask any early treatment effect and complicate the translation from preclinical research to clinical applications.

While differences between cancellous and cortical bone healing may exist, this study concentrates on the healing of cancellous bone within the defect. This animal model is developed to improve future investigations of bone graft substitutes that would interact with cancellous bone. Therefore, it is important to appropriately characterize this region.

In this pilot study, three different defect diameters of fixed depth were compared in both 18-month-old and 5-year-old sheep. This study was undertaken to investigate the influence of age and defect size on bone healing. These are important parameters to consider when planning a preclinical investigation into bone grafts and their substitutes. This study is intended to provide rationale for selection of these parameters as well as a reference for the healing capability in this model without intervention. Additionally, this study demonstrates a clearly defined methodology for the quantification of the healing response and bone formation.

## Materials and Methods

Approval of the University of New South Wales Animal Care and Ethics Committee was obtained prior to the start of the study. Four sheep (18 months old and 5 years old) were used for this study. All animals were acclimatized for 7 days prior to the surgery. Sedation was induced using Zoletil (12 mg/kg) via an intramuscular injection followed by gaseous induction and maintenance using isoflurane (2–3%) and O_2_ (3 L/min). Fluids (Hartmanns, 1 L) were delivered intravenously throughout the procedure via a cannula. All animals received cephalothin (0.024 mg/kg IV) and benacillin [0.122 mL/kg intramedullary (IM)] after induction as antibiotic prophylaxis. Animals also received Temgesic (buprenorphine, 0.005 mg/kg IM) and Carprofen (4 mg/kg SC) for pre-emptive pain relief prior to the surgery.

On both hind limbs, a 6–8 cm longitudinal incision was made from 1 cm proximal to the medial femoral epicondyle and run distally. Diathermy was used on the fascial tissue to expose the joint capsule and bone. Defects were allocated across the sites and drilled to 25 mm deep with diameters of 8 (*n* = 6), 11 (*n* = 6), and 14 mm (*n* = 4) being placed using a pneumatic drill in the cancellous bone in the medial distal femoral epiphysis and medial proximal tibial epiphysis.

The 8 and 11 mm defects were placed with cannulated acorn drill bits, while the 14 mm defect was placed with a 14 mm hole-saw with 1/4″ pilot bit. An initial pilot hole was created prior to using the 14 mm hole-saw to maintain alignment when using the hole-saw. The defects were irrigated with saline during defect creation to act as a lubricant and coolant. The defect sites were cleaned with gauze and flushed with saline to remove residual bone particulate within the defect. The soft tissue was closed with 2–0 absorbable suture, and skin closed with 3–0 suture. Animals received carprofen (2–4 mg/kg) orally once per day for 7 days post-operatively and an IM injection of Temgesic (0.005 mg/kg) once per day for 3–5 days post-operatively. No protective devices were used during the post-operative period, and the animals were free to move in the pen. At 4 weeks post-operatively, the animals were euthanized by lethal overdose of sodium pentobarbitone (lethabarb, 1 mL/2 kg).

Harvested sites were radiographed at 4 weeks and graded on a 1–4 scale by three separate graders according to the radiographic grading scale (Table 1).

**Table 1:** **Radiographic grading scale**.

1	**No healing:** No visible bone formation within defect; radiographic density considerably less then adjacent bone
2	**Partial healing:** Some bone bridging with gaps; defect visible with radiographic density significantly less than adjacent bone.
3	**Partially complete healing:** Bone bridging evident with minimal gaps; defect visible with radiographic density approaching density of adjacent bone.
4	**Complete healing:** Bone bridging with no gaps; defect barely visible with radiographic density equal to adjacent bone.

Each defect was scanned on a μCT scanner (ANSTO, Sydney, NSW, Australia) with a resulting slice thickness of 110 μm. Using μCT software (Inveon Research Workplace, Siemens, Germany), semi-quantification of the bone volume to total volume (BV/TV) within each defect was achieved through selective intensity thresholding.

Each defect was sagittally cut along the 25 mm depth into four blocks, fixed with 10% buffered formalin, dehydrated, and PMMA embedded. Forty micrometer sections were cut from each block using a saw microtome (Leica SP1600, Leica Microsystems, Germany). Sections were stained with basic fuschin and methylene blue for histological analysis. The sections from the second block, 8–11 mm deep from the medial surface, were used for histomorphometric assessment of the percentage area of new bone formation and for bone ingrowth measurements.

Statistical analysis was performed using SPSS (IBM SPSS Statistics 20). To account for treatment clustering within animals, linear mixed-effects modeling was used to analyze the μCT and histomorphometric data, while generalized linear mixed modeling was used for the ordinal radiographic grading data. Bonferroni correction was used for multiple pairwise comparisons to reduce the possibility of type I error. For linear regression, Pearson product–moment correlation coefficients were computed. For all tests, a *p*-value of <0.05 was regarded as statistically significant. Otherwise, *p*-values are reported. Data are presented as the mean (±SE).

## Results

### Macroscopic evaluation

The animals tolerated the surgery well with no adverse events within the 4 weeks. Based on macroscopic evaluation, none of the defects appeared to have achieved pre-operative bone content at the defect site within the 4 weeks.

### Radiographic grading

**Figure 1:**
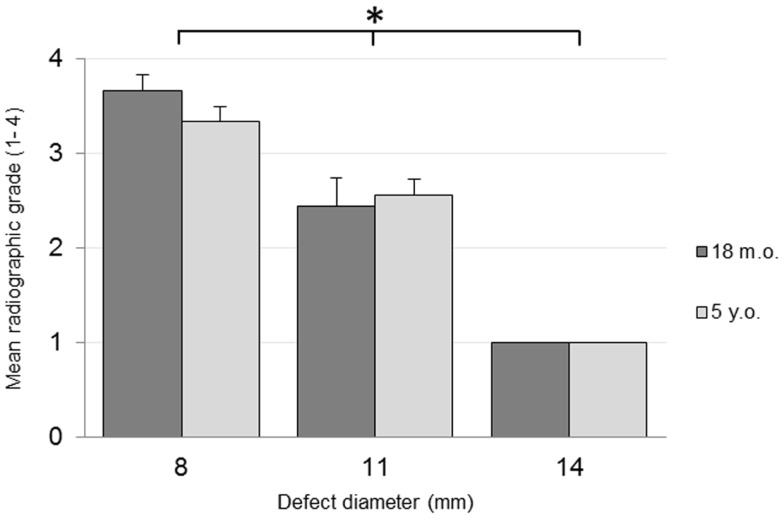
**Radiographic grading on a scale of 1–4**.

**Figure 2:**
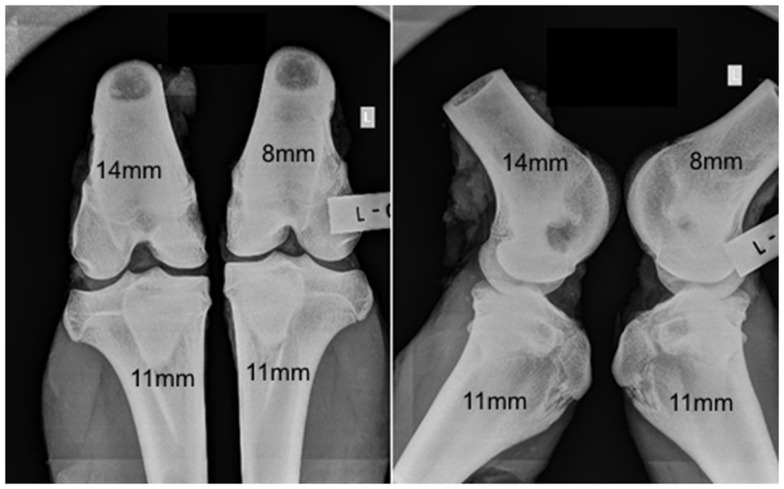
**Representative radiographs in anteroposterior (left) and lateral (right) orientations**.

No adverse reactions or issues were found radiographically. No significant effect was detected for the interaction of the animal age and defect diameter on the radiographic grade (*p* = 0.32), with both age groups having a similar changes in grade with an increasing defect diameter (Figure 1). Radiographic grades were statistically significant between all defect diameters with a combined-age mean grade of 3.5(±0.12) for the 8 mm defect, 2.5 (±0.17) for the 11 mm, and 1.0(±0.0) for the 14 mm defect. No statistically significant difference was detected between the two age groups (*p* = 0.58). Cortex bridging was not apparent in any of the defects based on radiographic assessment (Figure 2).

### μCT bone morphometry

**Figure 3:**
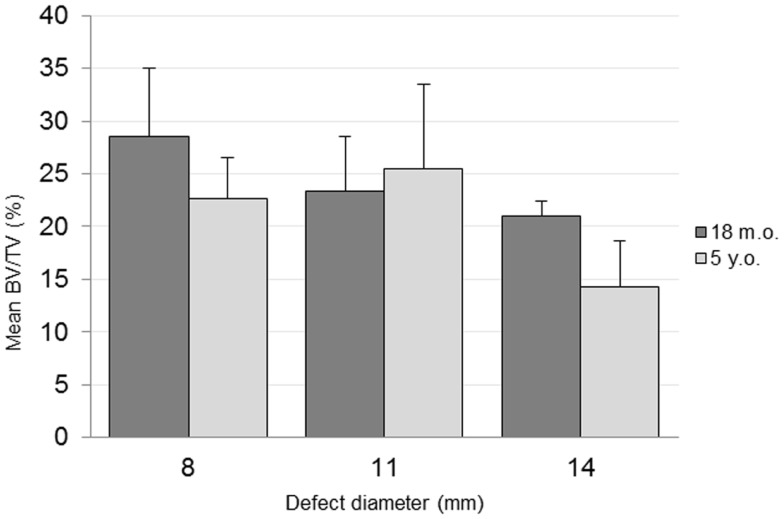
**μCT bone volume/total volume (BV/TV) quantification**.

**Figure 4:**
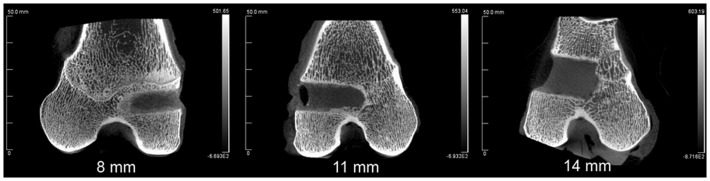
**Representative μCT of the three defect diameters in the distal femoral epiphyses**.

The combined-age mean BVtotal volume BV/TV was 25.6(±3.6)% for the 8 mm, 24.4(±4.3)% for the 11 mm, and 17.7(±2.2)% for the 14 mm defect (Figure 3). While a trend was apparent, none of the pairwise comparisons were statistically significant. Using this method and sample size, neither age (*p* = 0.62), defect diameter (*p* = 0.14), or their interaction (*p* = 0.65) were found to have a statistically significant effect on the μCT BV/TV. Interestingly, there was a positive linear correlation detected between TV and BV (*r* = 0.616, *n* = 16, *p* = 0.01), while no significant correlation was detected between the defect diameter and BV/TV (*r* = −0.342, *n* = 16, *p* = 0.20). Representative μCT slices are shown in Figure 4.

#### Histology

**Figure 5:**
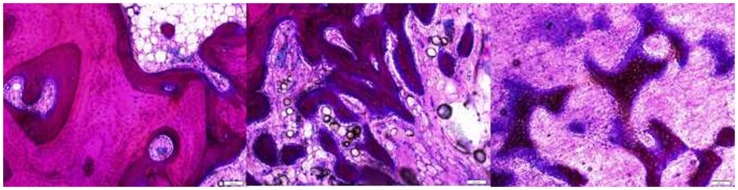
**The intact trabecule at the edge of the defect showed layers of new bone with lining of plump osteoblasts (left)**. The ingrowth bone adjacent to the surrounding bone showed cellular trabecule with irregular collagenous matrix and plump osteoblast linings (middle). The ingrowth bone deeper in the defect was formed with proliferating osteoblasts with minimal matrix (right).

**Figure 6:**
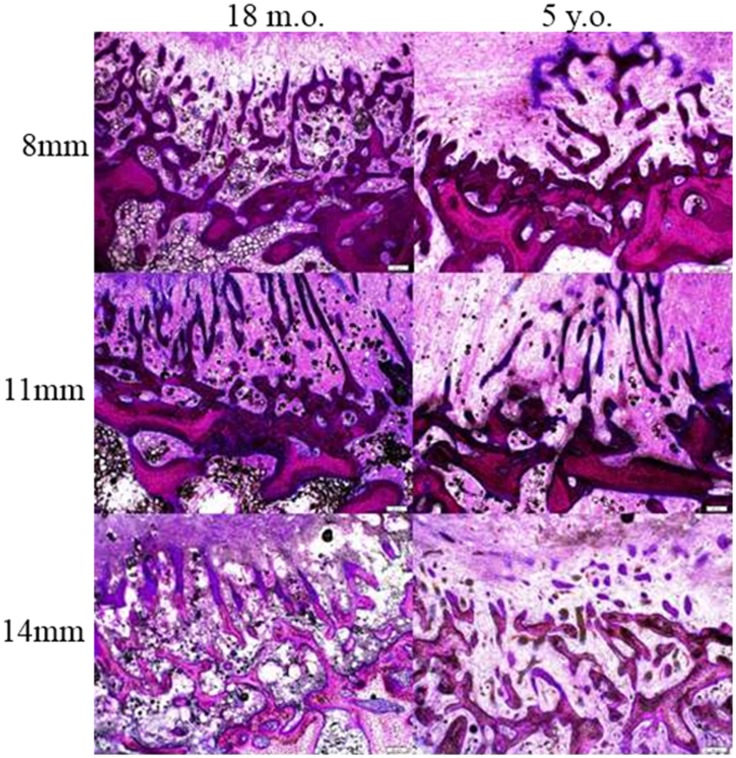
**Bone ingrowth of the defects at three different defect sizes in the two different ages of sheep**.

Cross sections of the defect at 8–10 mm deep from the medial surface were used for histological analysis. In general, the defects were filled with fibrous tissues with bone ingrowth from the surrounding trabecule. No inflammatory or foreign body reactions were noted in any cases. Layers of woven bone formed from the intact trabecule at the edge of the defect with a lining of plump osteoblasts. Bone ingrowth showed two features. The ingrowth bone adjacent to the surrounding bone showed high cellularity and an irregular collagenous matrix with a lining of plump osteoblasts. When the ingrowth progressed deeper into the defects, it was characterized by proliferating osteoblasts with minimal matrix (Figure 5). At the center of defects, only fibrous tissue was present. The defects from the two different age groups and three different sizes showed a similar pattern of healing (Figure 6), however, the amount of ingrowth bone, the depth of bone ingrowth, and the density of the fibrous tissues in the defects varied.

The depth of bone ingrowth was measured at eight evenly distributed points around the defect using the program of the DP72 camera (Olympus, Australia). The depth was consistently lower in the aged animals when compared with their young counterparts (Table 2); however, the effect of age did not reach statistical significance (*p* = 0.115). Neither the defect diameter (*p* = 0.426) nor the interaction of age and defect diameter (*p* = 0.714) was found to have a significant effect on the bone ingrowth.

**Table 2:** **Result summary [mean (SD)]**.

		Defect diameter (mm)		
		8	11	14
Radiographic grade (1–4)	18 months	3.7 (0.2)	2.4 (0.3)	1 (0)
	5 years	3.3 (0.2)	2.6 (0.2)	1 (0)
μCT BV/TV (%)	18 months	28.6 (5.3)	23.3 (4.2)	21.1 (1.0)
	5 years	22.7 (3.1)	25.5 (6.5)	14.3 (3.1)
Histomorphometry new bone area (%)	18 months	25.4 (3.9)	18.1 (3.4)	12.5 (2.2)
	5 years	20.1 (2.4)	17.9 (1.9)	12.0 (0.5)
Bone ingrowth (μm)	18 months	1171 (196)	1303 (193)	1200 (243)
	5 years	698 (73)	1158 (44)	893 (254)

**Figure 7:**
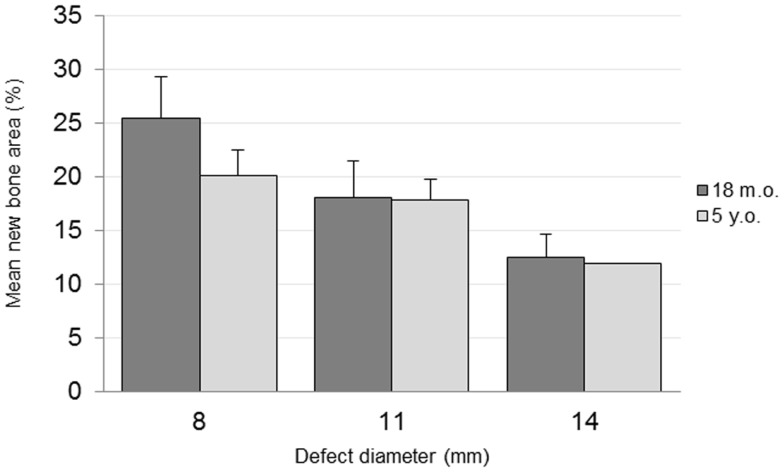
**New bone area quantification**.

**Figure 8:**
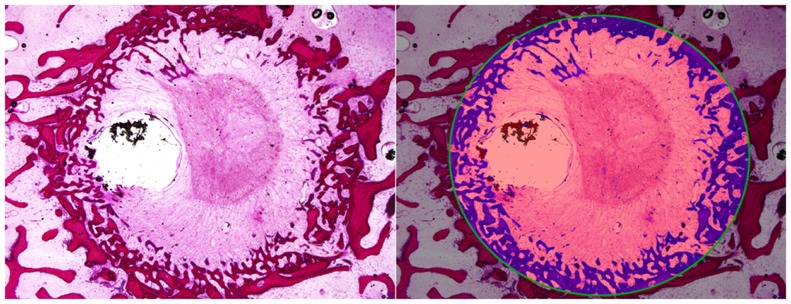
**Histomorphometric quantification of new bone within defect boundary at a cross section 8–10 mm deep from the medial surface**.

The percentage area of newly formed bone in each defect (BV/TV) was analyzed using histomorphometric analysis with an in-house image analysis program (Figures 7 and 8). Neither the age (*p* = 0.51) nor the interaction of age and defect diameter (*p* = 0.72) was found to have a significant effect on the histomorphometric new bone area. The combined-age mean new bone area was 22.8(±2.5)% for the 8 mm, 18.0(±2.0)% for the 11 mm, and 12.2(±1.1)% for the 14 mm defect, though the effect of the defect diameter did not reach statistical significance (*p* = 0.06).

## Discussion

Many animal models currently exist to study bone healing, ranging from bone window models utilizing bone chamber implants (4) to segmental defects attempting to replicate clinical situations (5, 6). This study used a relatively simple large animal model, placing confined defects within cancellous bone. A common alternative large animal model involves osteotomy to create a segmental bone defect (6, 7), though, as opposed to the confined model presented here, often requires fixation to stabilize the defect. Additionally, only one segmental defect is generally created per animal, often at the tibial diaphysis of sheep (5), limiting the available treatment sites per animal. Cancellous bone defects in either the tibial or femoral epiphyses have been previously reported (8, 9); however, while multiple sites in both the tibial and femoral cancellous bone have been previously reported (10), their characterization and use for bone healing is less well described. For this study, the 11 mm diameter defect is comparable to the cancellous bone defect of approximately 10 mm in sheep reported previously (2). The 8 and 14 mm diameters were selected as ±3 mm from this 11 mm diameter to investigate the effect of defect size.

### Age

An animal’s age generally has an impact on the healing process with the time required for healing largely increasing with age (11) and skeletal maturity (3). This delay in bone formation may be due to different temporal expression in aged animals, with delayed cellular differentiation and maturation possibly effecting bone formation (12). This pilot study was unable to find any significant effect from the animal’s age; however, the sample size is likely to have been a factor in this outcome. Objectively, the effect on bone healing from 3 mm increases in defect diameter was greater than the healing difference between 18-month-old and 5-year-old sheep. In a similar study comparing the effect of mechanical stability and age, Strube et al. (13) interestingly reported a significant interaction effect on the torsional stiffness of osteotomized rat femurs after 6 weeks. In their study, no significant differences were found between fixation in aged rats, whereas, a significant difference was reported for younger rats. No interaction effect between defect diameter and age was found in this study, suggesting that regardless of age, the increase in diameter results in less complete healing using this model.

On occasion, defects were found to intersect the tibial growth plate in the 18-month-old animals with bone replacing the cartilage zone. The high expression of TGF-β isoforms in this proliferating and hypertrophic chondrocyte region (14) may alter the signaling dynamics at the defect site, and the intersection of the growth plate in skeletally immature animals may confound the results in any future studies using the model. The unpredictable incidence of this issue can be avoided by using skeletally mature sheep in which the growth plate has closed.

### Defect diameter

The bone healing of the defects was delayed with an increasing defect diameter with an associated larger volume of fibrous tissue occupying the central region. An inadequate blood supply has been shown to induce greater fibrous tissue formation in an ischemic delayed-union model in mice (15), and a larger fibrous tissue volume in this model may present a larger obstacle for angiogenesis, resulting in slower rate of bone healing with increasing diameter.

While the defect diameter increments were 3 mm, this corresponded to substantially different defect volumes. The volumes of the defects were approximately 1.25 cc for the 8 mm diameter, 2.38 cc for the 11 mm diameter, and 3.85 cc for the 14 mm defect. A positive correlation was found between the total defect volume and new bone volume, suggesting a proportional relationship between the severity of the injury and the biological response. An array of signaling molecules regulate this biological response via ligand binding to receptor expressing cells at the injury site (16). Consequently, the increased internal surface area of larger defects may result in an increased surface area for cell surface receptor expression, resulting in the increased absolute new bone volume. This was illustrated with all empty defects healing via creeping substitution. Additionally, larger defect voids would increase the stress on the surrounding bone, and this may also partly account for the increase in the absolute new bone volume found within larger defects. For this reason, the bone healing capacity of this model may be further characterized by the total defect volume rather than solely the defect diameter. Using a similar model in sheep, Ding et al. (17) compared treatments after 9 weeks using 10 mm diameter, 11 mm deep implants into the medial and lateral femoral condyles bilaterally, with a reported defect void of 0.715 cc. In comparison, the 8 mm diameter defect in this model had a relatively high healing response, while being approximately twice the volume at 1.25 cc, and less than half the time at 4 weeks. Empty defects, such as presented in this study, are essential comparison groups to ensure new bone was truly a result of the intervention. The data from this study alludes that the defect volume has a substantial effect on the volume of new bone within the defect. Furthermore, relatively small defect volumes may not be sufficient to amplify differences in experimental groups effectively and this feature is an important consideration for future research considering this model.

Acute healing may result in complete healing without intervention. While the quantitative BV/TV of the 8 and 11 mm diameters were not significantly different with this low sample size, the 11 mm diameter is preferable over the 8 mm in both young and aged animals. This preference is due to the increased defect volume, larger fibrous tissue obstruction, lower BV/TV, and comparative lack of complications. However, as none of the defects had healed during the 4 weeks, all defect diameters could be potentially used to study cancellous bone healing at this time point.

An appreciable limitation in this model appeared during defect placement trails, where the 14 mm defect was only able to be placed in the cancellous bone of the femoral epiphysis; the tibial epiphysis did not have sufficient cancellous bone between the tibial plateau and the IM canal. While the results indicate that the 14 mm provides a significantly different environment than the smaller diameters, only two 14 mm defects can be placed per animal, as opposed to four 8 or 11 mm defects per animal. Hence, the preference for the 11 mm diameter defects.

### Surgical aspects

The tibial IM canal may cause two complications in this model. Firstly, the IM canal is a direct source of bone marrow derived stem cells (BMSC), and while BMSC may not spontaneously stimulate bone repair directly (18), the potential of BMSC to differentiate to osteoblasts and chondrocytes may confound the results if the IM canal is entered. Secondly, the effective use of this model requires all defects to be confined to ensure that graft material is contained within the defect volume. Therefore, care should be taken when placing the tibial defects to ensure the defect axis runs parallel to the tibial plateau and does not enter the IM canal.

The medial collateral ligament, originating from the medial femoral epicondyle region and inserting on the medial tibial metaphysis (19), may be unavoidably damaged during defect placement. This instability of the joint may contribute to patella subluxation, and appropriate muscle and skin closure is required to support patella stability. Additionally, the surgical approach should aim to minimize exposure of the bone surfaces to avoid excessive soft tissue damage, as this may lead to further instability of the tissues supporting the patella’s location.

The surgical creation of 11 mm diameter defects in the proximal tibial and distal femoral epiphyses of aged, skeletally mature sheep presents as a suitable large animal model to study early healing of cancellous bone defects. This refined model allows for the creation of four separate non-healing defects within a single sheep, and allows for detailed analysis using a variety of qualitative and quantitative histological and radiographic endpoints. This multi-site approach can reduce the animal numbers required to obtain information, and may be particularly suitable to compare multiple experimental treatment groups at once.

## Conflict of Interest Statement

The authors declare that the research was conducted in the absence of any commercial or financial relationships that could be construed as a potential conflict of interest.
